# Modulation of Apolipoprotein D levels in human pregnancy and association with gestational weight gain

**DOI:** 10.1186/1477-7827-7-92

**Published:** 2009-09-02

**Authors:** Sonia Do Carmo, Jean-Claude Forest, Yves Giguère, André Masse, Julie Lafond, Eric Rassart

**Affiliations:** 1 Centre de Recherche Biomed, Département des Sciences Biologiques, Université du Québec à Montréal, Montréal, Québec, H3C 3P8, Canada; 2 Hôpital Saint-François d'Assise, Centre Hospitalier Universitaire de Québec, Québec, Québec, G1R 2J6, Canada; 3 Hôpital Saint-Luc, Centre Hospitalier de l'Université de Montréal, Montréal, Québec, H2L 4M1, Canada; 4 Institut Santé-Société, Université du Québec à Montréal, Montréal, Québec, H3C 3P8, Canada

## Abstract

**Background:**

Apolipoprotein D (ApoD) is a lipocalin involved in several processes including lipid transport, but its modulation during human pregnancy was never examined.

**Methods:**

We investigated the changes in the levels of ApoD in the plasma of pregnant women at the two first trimesters of gestation and at delivery as well as in the placenta and in venous cord blood. These changes were studied in 151 women classified into 9 groups in relation to their prepregnancy body mass index (BMI) and gestational weight gain (GWG).

**Results:**

Plasma ApoD levels decrease significantly during normal uncomplicated pregnancy. ApoD is further decreased in women with excessive GWG and their newborns. In these women, the ApoD concentration was tightly associated with the lipid parameters. However, the similar ApoD levels in low cholesterol (LC) and high cholesterol (HC) women suggest that the plasma ApoD variation is not cholesterol dependant. A tight regulation of both placental ApoD transcription and protein content is most probably at the basis of the low circulating ApoD concentrations in women with excessive GWG. After delivery, the plasma ApoD concentrations depended on whether the mother was breast-feeding or not, lactation favoring a faster return to baseline values.

**Conclusion:**

It is speculated that the decrease in plasma ApoD concentration during pregnancy is an adaptive response aimed at maintaining fetal lipid homeostasis. The exact mechanism of this adaptation is not known.

## Background

Human pregnancy is associated with profound changes in the maternal lipid, carbohydrate and protein metabolisms. These changes are aimed at favoring the maintenance of pregnancy, sustaining fetal growth and brain development, and facilitating parturition [1]. Maternal metabolism is intimately linked with prepregnancy body mass index (BMI) and gestational weight gain (GWG), which greatly influence the outcome of pregnancy [2]. In association with an unhealthy lifestyle, suboptimal prepregnancy BMI and GWG also increase the risk of birth defects and chronic health problems in the children [3].

The maternal lipid metabolism during pregnancy is characterized by progressive increases in plasma cholesterol and triglyceride levels accompanied by increases in low density lipoprotein (LDL) and very low density lipoprotein (VLDL), leading to maternal hyperlipidemia during late pregnancy [4]. Cholesterol is essential for optimal embryonic and fetal development. It is an important component of plasma membranes, required for cell proliferation, differentiation and morphogenesis modulation [5]. It is also used by the placenta for the synthesis of steroid hormones [6]. Triglycerides are also crucial as they are used as a source of essential fatty acids for the fetus, although they do not directly cross the placental barrier [7].

Plasma lipid metabolism is regulated in part by the specific apolipoprotein constituents of the various lipoprotein classes. The pregnancy induced hyperlipidemia is accompanied by a rise in the plasma levels of some apolipoproteins, namely ApoA-1, ApoB, ApoC-II and ApoC-III. Apolipoprotein D (ApoD) is a secreted lipocalin assigned with many putative functions including lipid transport. Its macromolecular distribution extends from VLDL to very high density lipoproteins (VHDL) with a maximum concentration in high density lipoproteins 3 (HDL_3_) [8]. It is considered an atypical apolipoprotein as both its structure and major sites of synthesis differ from the other apolipoproteins. Indeed, in human, ApoD is poorly expressed in the liver and intestine while it is highly expressed in adrenal glands, spleen, kidneys, pancreas, placenta, nervous system, lungs, ovaries and testes [[9], Rassart *et al*., unpublished results]. Several hydrophobic molecules were identified as its potential ligands including cholesterol [9], bilirubin [10], pregnenolone, progesterone, estrogens [11,12] and arachidonic acid [13].

ApoD expression is modulated in many situations (for review see [14,15]), namely cellular growth and differentiation [16-19], cancer [19,20], nervous system pathologies [21-24] inflammation [25,26] and oxidative stress [27-29]. ApoD is also involved in gestation and fetus development. In human, it is up-regulated in the endometrium during the window of uterine receptivity for embryonic implantation [30], is highly expressed in placenta [9] and is found in colustrum and milk [31]. Its levels are also increased in fallopian tubes and ovaries of gestating compared to non-gestating guinea pigs [32] but are decreased in lactating compared to virgin mouse mammary gland [33]. During mouse development, ApoD expression is selectively modulated from embryonic day 8.5 (E8.5) to birth [34] and in brain, its expression coincides with the period of active myelination and synaptogenesis [35]. The role of ApoD in embryogenesis is not limited to mammals as it is found in the yolk of the rapidly growing chicken oocyte, where it might transport lipids or regulatory molecules such as vitamin A and thyroid hormones [36,37], and expressed during late chicken embryogenesis [38].

In spite of the important role of ApoD in lipid transport, its expression in gestation-related tissues and during fetus development, and also despite numerous studies on other apolipoproteins, there is no available information concerning ApoD modulation throughout human pregnancy.

To address this question, we measured ApoD levels in plasma at each trimester of pregnancy, in cord blood, two months after delivery and in placenta. We also examined how ApoD levels are modified by factors affecting lipid metabolism such as maternal prepregnancy BMI, gestational weight gain and hypercholesterolemia.

## Methods

### Population

The women participating in the study were recruited at their first prenatal visit, before their tenth week of pregnancy, at the Clinique Fidès of Montréal (Montréal, QC, Canada) and at the Saint-Luc Hospital's Perinatology Service of the Centre Hospitalier de l'Université de Montréal (CHUM, Montréal, QC, Canada), from 2002-2006. This study included 151 pregnant women of similar socio-economic situation. The study was approved by the ethical committee of CHUM and Université du Québec à Montréal (Montréal, QC, Canada). All subjects provided written informed consent. The exclusion criteria were gestational or type 2 diabetes, preeclampsia, hypertension, consumption of drugs interfering with lipid metabolism and complications during pregnancy or at delivery. A group of 5 non-pregnant women of similar age than the pregnant subjects examined, having a BMI of 20-26 kg/m^2 ^and with plasma biochemistry within the expected normal ranges was also included.

### Population classification

The study population was classified into 9 groups according to their prepregnancy BMI, confirmed at their first prenatal visit, and GWG. In accordance with Health Canada recommendations (2002), the normal values for BMI were established at 20-26 kg/m^2^. In order to facilitate the analysis, the normal values for GWG were established at 11-18 kg independently of the BMI [39-41]. The normal control group was defined as: BMI 20-26 kg/m^2^, GWG 11-18 kg. Women with a BMI under 20 or above 26 kg/m^2 ^were designated as low and high BMI, respectively, while women with a GWG under 11 or above 18 kg were designated as low and high GWG, respectively. A classification based on the median plasmatic total cholesterol level at delivery (6,85 mM) was also established. Women with a cholesterol concentration inferior to 7 mM (n = 38) were referred to as the low cholesterol group (LC) while women with a cholesterol concentration superior to 8 mM (n = 29) were considered as the non-pathologic high cholesterol group (HC) [42-44].

### Blood and tissue samples

Blood samples were collected at first (10-17 weeks) and second (22-28 weeks) trimesters of gestation and at delivery (36-40 weeks). Newborn blood samples were retrieved from the umbilical cord (venous cord blood). Blood samples were also collected two months after parturition (postpartum) from lactating and non-lactating women. The blood samples were centrifuged 15 min at 3,500 × g and plasma samples were kept at -20°C until analysis. The placentas, from vaginal delivery, were obtained from the Saint-Luc Hospital of CHUM (Montréal, QC, Canada) and were immediately immersed in Dulbecco's Modified Eagle Medium (DMEM) (Sigma, Oakville, ON, Canada) supplemented with antibiotics (penicillin, streptomycin and neomycin, Invitrogen, Burlington, ON, Canada) and NaHCO_3_. After the removal of the amnion, the chorion and the decidual layer, the placental tissue was randomly cut in 5 cm^2 ^sections, immediately frozen in liquid nitrogen and kept at -80°C until analysis.

### Plasma biochemistry

The plasma levels of total cholesterol, LDL-cholesterol, HDL-cholesterol and triglycerides were individually measured using the UniCel 36 DX600 Synchron Clinical System (Beckman-Coulter, Mississauga, ON, Canada) at the Clinical Biochemistry Service of Saint-François d'Assise Hospital (Québec, QC, Canada). Total plasma fatty acids were measured by gas phase chromatography. ApoA-1 and ApoB-100 levels were measured by RIA or ELISA methods at the Centre Hospitalier Universitaire de Québec (Québec, QC, Canada). ApoD levels were measured by competitive ELISA using the human ApoD monoclonal antibody 2B9 as previously described [22]. Briefly, microtiter plates were coated with antigen (1 μg ApoD/ml) overnight at 4°C in 5 mM glycin buffer (pH 9.2). Unreacted sites were blocked with PBS-BSA 1% for 1 h. A mixture containing diluted plasma and 2B9 antibody in PBS-BSA 1% then replaced the saturation solution. Bound 2B9 antibody was detected by peroxidase labeled anti-mouse IgG (KPL, Gaithersburg, MD) and revealed with ABTS substrate (2,2'-azino-bis(3-ethylbenzthiazoline-6-sulphonic acid; KPL). Optical density was measured at 410 nm. All quantifications were performed in triplicate.

### RNA isolation and quantitative Real-Time PCR (qRT-PCR)

Total RNA was prepared from 25 mg of placental tissue using the High Pure RNA Tissue Kit (Roche Diagnostics, Laval, QC, Canada) according to the manufacturer's instructions. Subsequently, cDNA was obtained by reverse transcription of total RNA using the Omniscript RT kit (Qiagen, Mississauga, ON, Canada). The quantification of ApoD and 18S transcripts were assessed by qRT-PCR using the LightCycler 480 SYBR Green I Master and the LightCycler 480 instrument and software (Roche Diagnostics). For each gene, diluted amounts of known templates provided quantitative standard curves from which cDNA copy number in clinical samples could be determined. ApoD transcript expression was then normalized versus the housekeeping gene 18S. The following primers were used in qRT-PCR: ApoD forward, 5'-CAT CTT GGG AAG TGC CCC AA-3'; ApoD reverse, 5'-CCA TCA GCT CTC AAC TCC TGG TTT-3'; 18S forward, 5'-CGC CGC TAG AGG TGA AAT TC-3'; 18S reverse, 5'-TTG GCA AAT GCT TTC GCT C-3'.

### Placental ApoD quantification

Placental tissues were rinsed 3-4 times in cold 0,9% NaCl solution containing a mixture of anti-proteases (Complete protease inhibitors-EDTA free, Roche Diagnostics) to remove blood from tissue. Placental tissues (1 g) were then homogenized in 1 ml cold lysis buffer (125 mM Tris-HCl, pH 8,0, 2 mM CaCl_2_, 1,4% (v/v) Triton X-100, 2% Complete protease inhibitors-EDTA free, 1 mM phenylmethylsulphonylfluoride) using a Polytron tissue homogenizer 3000 (Brinkman, Westbury, NY, USA). The homogenates were centrifuged at 10,000 × g for 25 min at 4°C. The protein concentration of the supernatant was determined using the BCA (bicinchoninic acid) assay (Pierce, Brockville, ON, Canada). Supernatants were kept at -80°C until analysis. The ApoD levels were measured by competitive ELISA as described above.

### Statistical analyses

Data were expressed as means ± SEM and analyzed with unpaired Student's *t*-test or Mann-Whitney U test. Results were considered significant at p < 0.05. The association between two variables of the same population was assessed using Pearson's correlation coefficient. All statistical analyses were performed using Prism 4.0 (GraphPad Software, San Diego, CA, USA).

## Results

### Population characteristics

As shown in Additional file 1, the 151 pregnant women participating to this study were classified into 9 groups according to their prepregnancy BMI and GWG. It was first observed that GWG had a greater influence on group characteristics at delivery than BMI. For example, in the high BMI group, suboptimal GWG, i.e. inferior or superior to the normal range of 11-18 kg, significantly reduced the pregnancy duration (Additional file 1). Nevertheless, only the normal BMI, low GWG group had smaller newborns (Additional file 2). Suboptimal GWG had an even larger impact on lipid parameters, mainly in the high BMI but also in the normal BMI group. Unexpectedly, however, both low and high GWG resulted in decreased lipid levels (Additional file 1). These changes however had a low incidence on the newborn lipid parameters (Additional file 2).

### Plasma ApoD levels decrease during pregnancy

**Figure 1:**
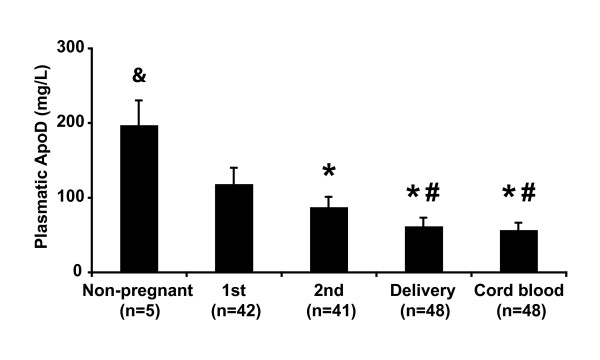
**Plasma ApoD levels during pregnancy**. ApoD levels were measured in women with a BMI of 20-26 kg/m^2 ^and a GWG of 11-18 kg at the two first trimesters of pregnancy (1^st ^and 2^nd^) and at delivery. ApoD levels in the venous cord blood were also measured. Data are expressed as means ± SEM. * indicates significant differences compared to the first trimester (p < 0.001). # indicates significant differences compared to the second trimester (p < 0.001). & indicates significant differences compared to the pregnant groups and to cord blood (p < 0.001).

Plasma ApoD levels were measured in women at each trimester of pregnancy. In the control group (BMI 20-26 kg/m^2^, GWG 11-18 kg), plasma ApoD concentration declined early in pregnancy as its levels were already reduced by 40% at the first trimester of pregnancy compared to non-pregnant women (Figure 1). ApoD levels continued to progressively decrease at the second trimester and until delivery where they reached 30% of the levels in non-pregnant women (Figure 1). A similar significant decrease of ApoD during gestation was also observed in our 8 other groups (data not shown). Interestingly, ApoD levels in the venous cord blood were similar to the levels in the mother plasma at delivery in all groups, except for the normal BMI (20-26 kg/m^2^), low GWG (< 11 kg) group where cord blood had inferior ApoD levels than the mother's blood (Figure 1 and Table 1). In spite of this, by using the Pearson's correlation coefficient, no correlation was found between ApoD plasma levels in the mother and the venous cord blood (Table 2 and Additional file 3).

**Table 1:** Plasma ApoD (mg/L) in the mother at delivery and in the cord blood.

MOTHER			
	BMI (kg/m^2^)		
GWG (kg)	< 20	20-26	> 26
< 11	76.08 ± 31.36 (n = 9)	87.84 ± 21.94 (n = 28)	64.86 ± 21.54 (n = 20)
11-18	59.30 ± 16.65 (n = 14)	61.69 ± 10.93 (n = 48)	54.44 ± 11.30 (n = 18)
> 18	25.99 ± 14.02 (n = 4) * †	27.45 ± 9.10 (n = 6) * †	46.44 ± 20.96 (n = 4)

CORD BLOOD			
	BMI (kg/m^2^)		
GWG (kg)	< 20	20-26	> 26

< 11	60.35 ± 21.12 (n = 9)	**43.48 ± 8.73 (n = 28) **#	67.28 ± 17.20 (n = 20)
11-18	64.61 ± 22.20 (n = 14)	56.63 ± 8.96 (n = 48)	85.06 ± 70.11 (n = 18)
> 18	20.26 ± 8.56 (n = 4) * †	36.98 ± 13.00 (n = 6) *	**17.25 ± 6.51 (n = 4) **†

Data are organized according to maternal weight gain during pregnancy (GWG) and body mass index (BMI) at first trimester. Data are expressed as mean ± SEM. * Groups statistically different (p < 0.01) from the normal GWG group (11-18 kg) of the same BMI. **† **Groups statistically different (p < 0.01) from the low GWG group (< 11 kg) of the same BMI. A difference between mother and cord blood of the corresponding group (p < 0.001) is indicated by **#**. All groups presenting differences are in bold characters.

**Table 2:** Association of maternal plasma ApoD levels with clinical and biochemical characteristics at delivery in relation to GWG.

	GWG < 11 kg n = 57		GWG 11-18 kg n = 80		GWG > 18 kg n = 14	
	Mean ± SEM	*r*	Mean ± SEM	*r*	Mean ± SEM	*r*
Mother age (years)	31.70 ± 0.62 †††	0.098	30.37 ± 0.45	0.085	29.63 ± 1.07	0.241 ***
Gestational age (weeks)	38.96 ± 0.17 †††	-0.065	39.40 ± 0.14	0.137	39.32 ± 0.37	-0.414 ***
BMI (kg/m^2^)	25.39 ± 0.61 †††	-0.077	23.65 ± 0.37	-0.044	24.37± 1.59	0.232 ***
GWG (kg)	8.08 ± 0.30 †††	0.303 ***	14.16 ± 0.21	-0.172	22.75 ± 2.56 †††	-0.365 ***
Newborn weight (g)	3256.96 ± 53.01 †††	-0.300 **	3411.22 ± 48.54	0.126	3407.16 ± 114.40	-0.552
Newborn height (cm)	51.02 ± 0.28	-0.166	51.55 ± 0.22	0.107	52.00 ± 0.54	-0.559 ***
Cord blood ApoD (mg/L)	54.43 ± 8.01	0.169	65.65 ± 9.35	0.135	28.04 ± 7.14 †††	-0.080
						
Total cholesterol (mM)	6.75 ± 0.21	0.039	7.04 ± 0.15	-0.153	6.25 ± 0.25 †††	-0.563 **
LDL-cholesterol (mM)	3.67 ± 0.17	0.035	3.90 ± 0.14	-0.094	3.28 ± 0.20 †††	-0.724 **
HDL-cholesterol (mM)	1.76 ± 0.06	0.171	1.81 ± 0.05	-0.145	1.61 ± 0.14 ††	-0.598 **
Triglycerides (mM)	2.88 ± 0.14	-0.124	2.94 ± 0.10	-0.085	2.95 ± 0.34	0.631 **
Free fatty acids (mmol/L)	0.66 ± 0.05	-0.107	0.65 ± 0.04	-0.175	0.60 ± 0.06	-0.358 **
ApoA-I (g/L)	2.12 ± 0.05	-0.003	2.14 ± 0.04	-0.215 ***	2.04 ± 0.09	-0.554 **
ApoB-100 (mg/L)	1.31 ± 0.05	0.027	1.40 ± 0.04	-0.165	1.27 ± 0.05 †††	-0.243 **
ApoD (mg/L)	77.63 ± 13.32		59.44 ± 7.52		31.75 ± 7.59 †††	

† p < 0.05, †† p < 0.01, ††† p < 0.001 compared to the GWG 11-18 kg group. Pearson correlations (*r*) are significant at * p < 0.05, ** p < 0.01, ***p < 0.001. Significant Pearson correlations are indicated in bold.

### Influence of prepregnancy BMI and GWG on plasma ApoD levels

Maternal prepregnancy BMI did not present an impact on plasma ApoD levels at delivery (Table 1). However, a GWG superior to 18 kg caused a decrease in maternal ApoD levels in the normal and low BMI groups but not in the high BMI group (Table 1). Similarly, GWG but not BMI also affected venous cord blood ApoD concentration. Newborns from mothers having a GWG superior to 18 kg had lower cord blood ApoD levels than newborns from the other groups (Table 1).

### Association between plasma ApoD levels and pregnancy parameters

The correlation between ApoD concentration at delivery and the other pregnancy-related parameters was explored using Pearson's correlation coefficient. In the control group, ApoD levels correlated only with total-cholesterol, in a positive manner (Additional file 3). In the other groups, ApoD correlations were different and specific to each group (Additional file 3). Then, since only GWG had an impact on ApoD values (Table 1), we created 3 new groups containing all women with similar GWG, regardless of their BMI (Table 2). In women with low GWG, ApoD values were directly correlated with GWG and inversely correlated with the newborn weight. Moreover, in women with normal GWG, ApoD was inversely correlated with ApoA-I. In contrast, in women with high GWG, which present lower ApoD levels, ApoD was correlated with most of the parameters. ApoD was strongly negatively correlated with most of the lipid parameters except triglycerides with which ApoD was directly correlated. ApoD was also negatively correlated with gestational age, GWG and newborn height and directly correlated with mother's age and BMI (Table 2). However, although many of the lipid parameters in women with high GWG are significantly different from the normal GWG group, all newborns displayed similar lipid parameters independently of maternal GWG (data not shown).

### ApoD and cholesterol

To verify if the low ApoD level in women with excessive GWG was attributable to their lower cholesterol levels (Additional file 1), and because of the correlation between plasma ApoD and cholesterol levels in the control group (Additional file 3), we compared the ApoD levels between low cholesterol (LC) and high cholesterol (HC) women (Table 3). The HC group had higher total and LDL-cholesterol, triglycerides, ApoA-I and ApoB-100 levels but lower GWG (Table 3). However, both groups displayed similar ApoD levels. The 2 groups were also different from the control group described in Additional file 1. As expected, the LC group had lower while the HC group had higher levels of total cholesterol, LDL-cholesterol and ApoB-100. In this aspect, the LC group was similar to the high GWG groups (Additional files 1 and 3). The HC group also showed lower GWG and higher triglycerides levels than the control group (Additional files 1 and 3). In spite of this, all newborns displayed similar lipid parameters independently of maternal cholesterol levels (data not shown).

**Table 3:** Population characteristics according to maternal plasma cholesterol levels at delivery and association with plasma ApoD levels.

	LC n = 38		HC n = 29	
	Mean ± SEM	*r*	Mean ± SEM	*r*
Mother age (years)	30.40± 0.78	0.282 ***	31.70± 0.91	-0.055
Gestational age (weeks)	39.30± 0.25	0.100	39.50± 0.23	-0.066
BMI (kg/m^2^)	22.68± 0.33	0.086	23.26± 0.68	0.175
GWG (kg)	14.26± 0.43	-0.321 ***	12.16± 0.47 † ***	-0.054
Newborn weight (g)	3452.43± 83.90	-0.329	3389.27± 67.13	0.398 *
Newborn height (cm)	51.86± 0.39	0.114	51.77± 0.40	0.009
Cord blood ApoD (mg/L)	59.17± 13.31	-0.005	59.33± 11.57	0.490
				
Total cholesterol (mM)	5.95± 0.13 †	-0.533 ***	9.08± 0.17 † ***	0.023
LDL-cholesterol (mM)	2.94± 0.12 †	0.314 ***	5.65± 0.18 † ***	-0.040
HDL-cholesterol (mM)	1.75± 0.09	-0.215 ***	1.79± 0.10	0.063
Triglycerides (mM)	2.76± 0.18	-0.178	3.59± 0.18 † ***	-0.033
Free fatty acids (mmol/L)	0.51± 0.06	-0.268 ***	0.63± 0.08	-0.310 ***
ApoA-I (g/L)	2.09± 0.06	-0.422 ***	2.24± 0.08 *	0.028
ApoB-100 (mg/L)	1.18± 0.04 †	-0.413 ***	1.82± 0.04 † ***	0.071
ApoD (mg/L)	57.69± 14.16		63.05± 11.54	

LC: low cholesterol; HC: high cholesterol. * p < 0.05, ** p < 0.01, *** p < 0.001 compared to the LC group. † p < 0.001 compared to the control group (BMI 20-26 kg/m^2^, GWG 11-18 kg). Pearson correlations (*r*) are significant at * p < 0.05, ** p < 0.01, *** p < 0.001. Significant Pearson correlations are indicated in bold.

The cholesterol level greatly affected the association of ApoD with maternal and newborn characteristics, ApoD being more associated with pregnancy-related characteristics in LC than in HC women (Table 3). These associations differed from the ones described for the control group (Additional file 3) but showed similarities to those in high GWG groups (Table 2).

### ApoD in placenta

**Figure 2:**
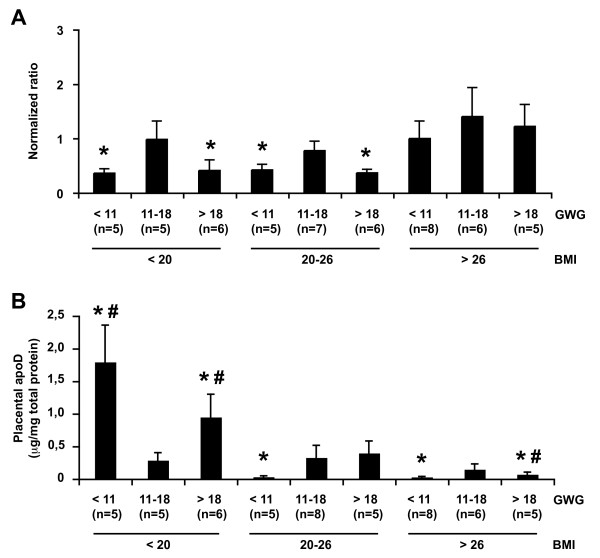
**ApoD levels in placenta according to pre-pregnancy BMI and GWG**. (A) The level of apoD mRNA expression was measured by qRT-PCR using specific primers for apoD. Data are expressed as the normalized ratio of apoD mRNA to 18S RNA (means ± SEM). (B) The placental ApoD protein content was measured by ELISA. The values (means ± SEM) were normalized by the total protein content. * indicates significant differences (p < 0.01) from the normal control group (BMI 20-26 kg/m^2^, GWG 11-18 kg). # indicates significant differences (p < 0.01) from the group of similar GWG and normal BMI (20-26 kg/m^2^).

Because of the high levels of ApoD mRNA in human placenta and the importance of this tissue in fetal development, we examined placental ApoD transcript and protein content in relation to maternal BMI and GWG. Besides its influence on plasma ApoD levels (Table 1), maternal GWG also affected placental ApoD transcription (Figure 2A). However, in contrast to plasma, ApoD transcript levels were decreased in both low and high GWG compared with normal GWG groups. This pattern was mostly noticed in women with normal or low BMI but, although not significant, this tendency was also observed in women with high BMI (Figure 2A). Interestingly, the placental ApoD protein content diverged from the mRNA levels and was affected both by GWG and by BMI (Figure 2B). High BMI with suboptimal GWG groups contained the lower levels of ApoD whereas the higher levels of ApoD were observed in low BMI groups having suboptimal GWG. Low levels of ApoD were also detected in the normal BMI, low GWG group. Nevertheless, ApoD levels were similar in normal GWG groups, independently of BMI.

### Postpartum plasma ApoD levels

**Figure 3:**
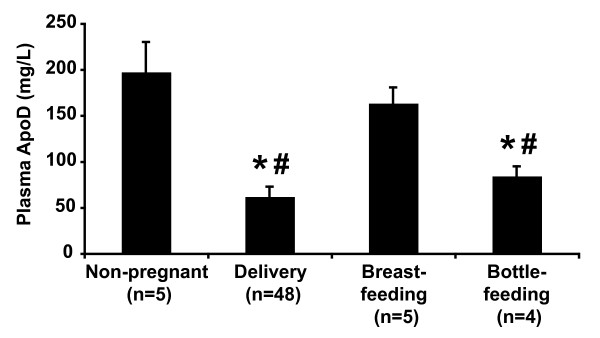
**The effect of breast- and bottle-feeding on plasma ApoD levels**. ApoD concentration was measured in mothers two months after delivery (women with a BMI of 20-26 kg/m^2 ^and a GWG of 11-18 kg) and compared with levels at delivery and in non-pregnant women (BMI 20-26 kg/m^2^). Data are expressed as means ± SEM. * indicates significant differences compared to non-pregnant women (p < 0.001). # indicates significant differences compared to breast-feeding women (p < 0.001).

Given that plasma ApoD levels were decreased by 70% at the end of pregnancy, we examined if, in control women, ApoD levels returned to baseline values two months after delivery and whether these levels were affected by lactation (Figure 3). Breast-feeding (lactating) women had plasma ApoD levels similar to non-pregnant women. However, in bottle-feeding (non-lactating) women, ApoD concentration remained comparable to the levels at delivery.

## Discussion

To our knowledge, this study is the first to demonstrate that plasma ApoD levels decrease during pregnancy. Plasma ApoD levels were already significantly lower during first trimester of pregnancy than values detected in non-pregnant women and continued to decrease until the end of pregnancy. The mechanisms governing this decrease are currently unknown, but many potential reasons could explain it.

As for albumin, which concentration is lowered by 30% during pregnancy [45], the decrease in plasma ApoD levels could be explained in part by hemodilution. Maternal blood volume increases until term and although dependant on many factors, the average increase in volume at term is 45-50% [46]. Still, although it could explain the decrease in plasma ApoD levels at the first and second trimesters of pregnancy, hemodilution alone cannot explain the observed 70% decrease of plasma ApoD at delivery.

Low levels of plasma ApoD could also result from decreased ApoD synthesis or secretion. Plasma ApoD can originate from many sources, one of which being the placenta. Since ApoD mRNA levels are almost as high in placenta as in adrenal glands and spleen, major sources of ApoD in non-pregnant humans [9], decreased ApoD transcription is unlikely the origin of low plasma ApoD concentration. Another argument against ApoD transcriptional downregulation is the presence of oxidative and inflammatory markers mostly in complicated but also in normal pregnancies [47]. Oxidative stress [26-29] and inflammation [25,26] were both reported as intensifying ApoD mRNA production. ApoD decrease could also be caused by the inhibition of its translation or the increase of its degradation rate. More likely, the decline in ApoD levels could be imputable to its reduced secretion by placenta and other tissues during pregnancy. In addition, as prolonged exposure to physiological concentrations of 17 beta-estradiol inhibited markedly (70-90%) ApoD secretion in human breast cells [19], high estrogen concentrations, as seen during gestation [48,49], could be the root of plasma ApoD decrease.

Assuming that ApoD synthesis and degradation rates remain unchanged, a reduced secretion will result in ApoD accumulation inside tissues. In the placenta, in collaboration with other molecules, ApoD could play a key role in the transport of essential nutrients, regulation factors and toxic metabolites between the mother and the fetus. Those could include cholesterol [9], arachidonic acid [13,50], steroids [11,12], vitamin A and thyroid hormones [37] as well as the removal of bilirubin [10].

It was already reported that ApoD levels in newborns represent about 37% of the levels found in adults [51,52]. Accordingly, we observed that the concentration of circulating ApoD in the venous cord blood was very similar to that of the mother at delivery, which is about 30% of the levels in non-pregnant women, although the levels found in the cord blood and in the mother were not statistically correlated. This lack of correlation suggests that ApoD does not pass through the placental barrier. Since ApoD mRNA expression occurs as early as E8.5 during mouse embryogenesis [34], the fetus might not be dependent on a maternal ApoD supply. Furthermore, the similarity in mother and venous cord blood ApoD concentrations suggests that even if maternal plasma ApoD does not directly cross the placental barrier, both might be subjected to the same regulatory mechanisms.

We also observed that, while prepregnancy maternal BMI had no incidence on plasma ApoD concentrations on both mother and cord blood, high GWG (> 18 kg) significantly decreased ApoD values. This decrease is very likely multifactorial. In this particular case, it could, among others, be related to defects in lipid metabolism. Indeed, high GWG groups displayed lower levels of total cholesterol, LDL-cholesterol, and ApoB-100. This was present in normal and high BMI groups but the tendency, although not significant, was also observed in the low BMI group. This was also observed in the group composed of all women with high GWG regardless of their BMI. In addition, in this group, ApoD and lipid markers were strongly correlated. An involvement of ApoD in lipid metabolism was already suggested. Because of its capacity to bind cholesterol and its presence on lipoprotein fractions [8], ApoD, in collaboration with ApoA-I, lecithin-cholesterol acyltransferase (LCAT) and cholesteryl ester transfer protein (CETP) [53], may participate in the cholesterol transport pathway. Furthermore, genetic variation in ApoD affects the serum lipid levels [54].

Nevertheless, lipid levels are probably not the major factors responsible for low ApoD levels in these groups. Indeed, high GWG groups are not the only ones presenting levels of total cholesterol, LDL-cholesterol, and ApoB-100 different from the control group as well as correlations between ApoD and lipid levels. In addition, the low BMI group does not show a significant decrease of these lipid parameters. Finally, the ApoD concentration is similar in the control, HC and LC groups, the latter showing similarities with the high GWG groups.

Our results also suggest that a tight regulation of placental ApoD transcription and secretion is probably involved in the lower circulating ApoD concentrations in women with excessive GWG. In the view that ApoD may play a key role in the control of fetal homeostasis, the regulation of ApoD levels becomes vital for the pregnancy outcome and the health of the newborn. Thus, in women with high GWG, a decrease in circulating ApoD is desirable in order to reduce lipid transfer to fetus. This could be achieved both by reducing the ApoD transcription and by preventing ApoD from leaving the placenta. Consistently, high GWG have low ApoD mRNA levels and normal or high placental ApoD content. According to this hypothesis, the lower production of ApoD in low GWG women is disadvantageous. It would then be compensated by an increased secretion or lower degradation by the placenta and possibly other tissues resulting in adequate ApoD protein concentration in the circulation. In overweight women (high BMI), this control is probably altered, as evidenced by the high ApoD mRNA and low ApoD protein levels in the placenta independently of the GWG. This may explain why the plasma ApoD levels are similar in high GWG and in the control group. Still, plasma ApoD levels are not solely dependent on placental regulation as high BMI groups, in spite of high ApoD mRNA and low ApoD protein levels in the placenta, did not show higher plasma ApoD concentration than the control group. Similarly, despite low ApoD transcript levels and high protein content in the placenta, the low BMI, low GWG group had plasma ApoD levels similar to the control group. This suggests that other tissues have the ability to secrete or trap ApoD and that they may not be subjected to the same regulatory pathways than the placenta.

After delivery, the return to basal, non-pregnant, plasma ApoD levels, such as observed in breast-feeding women was expected. However, the fact that bottle-feeding women still have the same plasma ApoD levels than at delivery is intriguing and suggests again that the regulation of ApoD levels is complex. A possible explanation of this discrepancy is that breast-feeding mothers have high levels of prolactin. Prolactin was reported to exert an antagonistic action on estrogen production in order to maintain lactation [48,55] and could thus favor ApoD production. In addition, breast-feeding induces the production of cytokines [56,57], which may contribute to the higher levels of circulating ApoD.

## Conclusion

Plasma ApoD levels decrease during normal uncomplicated human pregnancy. ApoD is further decreased in women with excessive GWG and their newborns. The cause of this decrease, controlled in part at the transcriptional level in the placenta, is probably multi-factorial and may be related to pregnancy-induced changes in lipids and hormone levels. Still, the connection between lipids, estrogen and apoD levels is speculative and requires further studies.

## Competing interests

The authors declare that they have no competing interests.

## Authors' contributions

SDC carried out the laboratory work, participated in conceiving and designing of the study, performed the statistical analysis and drafted the manuscript. J-CF, YG and AM were involved in data acquisition and analysis. ER and JL conceived and design the study, analyzed the results and edited the manuscript. All authors read and approved the final manuscript.

## Supplementary Material

Additional file 1 **Table 4: Population characteristics at delivery**. Plasma biochemistry of the mother at delivery organized according to maternal body mass index and gestational weight gain. Click here for file

Additional file 2 **Table 5: Newborn characteristics at birth**. Plasma biochemistry of the newborn at birth organized according to maternal body mass index and gestational weight gain. Click here for file

Additional file 3 **Table 6: Association of plasma ApoD levels with clinical and biochemical characteristics in women at delivery**. The data provided represent the Pearson correlations coefficients (r) between plasma ApoD levels and maternal characteristics at delivery. Click here for file
