# Supplementary material for: Modulation of Apolipoprotein D levels in human pregnancy and association with gestational weight gain
Source: Reprod Biol Endocrinol. 2009 Sep 2;7:92. doi: 10.1186/1477-7827-7-92 (PMC3224896; doi:10.1186/1477-7827-7-92)
Supplement: Additional file 1 — Table 4: Population characteristics at delivery. Plasma biochemistry of the mother at delivery organized according to maternal body mass index and gestational weight gain. [file 1477-7827-7-92-S1.doc]

**Table 4: Population characteristics at delivery.**

|  |  | | | |  | | |  | | | |
| --- | --- | --- | --- | --- | --- | --- | --- | --- | --- | --- | --- |
|  | BMI < 20 kg/m^2^ | | | | BMI 20-26 kg/m^2^ | | | BMI > 26 kg/m^2^ | | | |
|  | GWG < 11 kg  n= 9 | GWG 11-18 kg  n= 14 | GWG > 18 kg  n= 4 | GWG < 11 kg  n= 28 | | GWG 11-18 kg  n= 48 | GWG > 18 kg  n= 6 | | GWG < 11 kg  n= 20 | GWG 11-18 kg  n= 18 | GWG > 18 kg  n= 4 |
|  |  |  |  |  | |  |  | |  |  |  |
| Mother age (years) | 31.80±1.40 | 29.71±0.74 | 32.00±1.90 | 31.15±0.96 | | 30.49±0.61 | 27.89±1.33 | | 32.37±0.98 | 30.48±0.96 | 30.00±2.86 |
| Gestational age (weeks) | 39.10±0.62 | 39.21±0.39 | 40.17±0.54 | 39.20±0.23 | | 39.49±0.19 | 39.22±0.52 | | **38.59±0.25 *** | 39.32±0.21 | **38.25±0.85 *** |
| BMI (kg/m^2^) | 18.81±0.31 | 19.06±0.16 | 18.69±0.50 | 23.09±0.27 | | 22.66±0.22 | 22.90±0.54 | | 30.81±0.75 | 28.43±0.45 | 34.40±2.64 |
| GWG (kg) | 9.07±0.29 | 13.37±0.36 | 30.04±8.29 | 7.80±0.45 | | 14.19±0.28 | 20.21±0.57 | | 8.04±0.52 | 14.54±0.44 | 18.70±0.29 |
| Total cholesterol (mM) | 7.85±0.75 | 7.31±0.46 | 6.81±0.28 | 6.81±0.21 | | 6.99±0.19 | **6.25±0.36 *** † | | **6.26±0.39 *** † | 6.97±0.31 | **5.11±0.40 *** † |
| LDL-cholesterol (mM) | 4.69±0.62 | 4.25±0.44 | 3.70±0.32 | 3.79±0.19 | | 3.86±0.16 | **3.29±0.20 *** † | | **3.13±0.30 *** † | 3.75±0.29 | **2.41±0.53 *** † |
| HDL-cholesterol (mM) | 1.70±0.10 | 1.85±0.15 | 1.66±0.11 | 1.86±0.08 | | 1.83±0.06 | 1.68±0.30 | | 1.64±0.11 | 1.73±0.12 | **1.34±0.23 *** † |
| Triglycerides (mM) | 3.16±0.43 | 2.87±0.24 | 3.15±0.58 | **2.55±0.14 *** † | | 2.83±0.11 | 2.77±0.50 | | **3.25±0.29 *** | 3.24±0.28 | 2.96±1.00 |
| Free fatty acids (mmol/L) | **0.48±0.09 *** † | 0.66±0.09 | 0.64±0.12 | 0.70±0.08 | | 0.61±0.06 | 0.67±0.09 | | 0.67±0.08 | 0.73±0.06 | **0.36±0.06 *** † |
| ApoA-I (g/L) | 2.13±0.10 | 2.12±0.12 | 2.08±0.08 | 2.14±0.07 | | 2.16±0.06 | 2.11±0.18 | | 2.08±0.09 | 2.13±0.09 | **1.80±0.08 *** † |
| ApoB-100 (mg/L) | 1.53±0.14 | 1.42±0.10 | 1.39±0.04 | **0.93±0.06 *** † | | 1.38±0.05 | **1.27±0.05*** † | | **1.19±0.07 *** † | 1.42±0.09 | **1.04±0.14*** † |
|  |  |  |  |  | |  |  | |  |  |  |

Data are organized according to maternal body mass index (BMI) at first trimester of pregnancy and weight gain during pregnancy (GWG). Results are expressed as mean ± SEM. ***** Groups statistically different (p<0.01) from the normal control group (BMI 20-26 kg/m^2^, GWG 11-18 kg) and from the corresponding normal GWG group (11-18 kg) of similar BMI for a given parameter. All groups presenting differences are in bold characters. Differences in BMI and GWG were expected, as they served as classification criteria, and are not highlighted.
