# Supplementary material for: Modulation of Apolipoprotein D levels in human pregnancy and association with gestational weight gain
Source: Reprod Biol Endocrinol. 2009 Sep 2;7:92. doi: 10.1186/1477-7827-7-92 (PMC3224896; doi:10.1186/1477-7827-7-92)
Supplement: Additional file 2 — Table 5: Newborn characteristics at birth. Plasma biochemistry of the newborn at birth organized according to maternal body mass index and gestational weight gain. [file 1477-7827-7-92-S2.doc]

**Table 5: Newborn characteristics at birth.**

|  |  | | | | |  | | | |  | | |
| --- | --- | --- | --- | --- | --- | --- | --- | --- | --- | --- | --- | --- |
|  | BMI < 20 kg/m^2^ | | | | | BMI 20-26 kg/m^2^ | | | | BMI > 26 kg/m^2^ | | |
|  | | GWG < 11 kg  n= 9 | GWG 11-18 kg  n= 14 | GWG > 18 kg  n= 4 | GWG < 11 kg  n= 28 | | GWG 11-18 kg  n= 48 | GWG > 18 kg  n= 6 | GWG < 11 kg  n= 20 | | GWG 11-18 kg  n= 18 | GWG > 18 kg  n= 4 |
|  | |  |  |  |  | |  |  |  | |  |  |
| Newborn weight (g) | | 3220.00±133.95 | 3405.86±117.19 | 3554.17±209.46 | **3147.71±76.17 *** | | 3373.34±63.26 | 3375.89±159.79 | 3408.22±82.74 | | 3494.52±99.58 | 3259.25±302.11 |
| Newborn height (cm) | | 50.55±1.01 | 51.50±0.57 | 52.08±0.99 | **50.73±0.36 *** | | 51.70±0.29 | 52.11±0.79 | 51.57±0.44 | | 51.28±0.44 | 51.26±1.43 |
| Total cholesterol (mM) | | 2.06±0.27 | 1.69±0.11 | 1.67±0.31 | 1.74±0.08 | | 1.71±0.06 | 1.67±0.14 | 1.86±0.26 | | 1.90±0.20 | **2.43±0.54 *** |
| LDL-cholesterol (mM) | | 1.02±0.19 | 0.73±0.09 | 0.73±0.19 | 0.79±0.05 | | 0.75±0.04 | 0.68±0.08 | 0.86±0.15 | | 0.84±0.14 | 1.09±0.23 |
| HDL-cholesterol (mM) | | 0.74±0.08 | 0.70±0.05 | 0.71±0.15 | 0.71±0.04 | | 0.67±0.04 | 0.76±0.11 | 0.74±0.08 | | 0.76±0.06 | 1.00±0.39 |
| Triglycerides (mM) | | 0.68±0.21 | 0.58±0.09 | 0.51±0.12 | 0.52±0.07 | | 0.63±0.05 | 0.50±0.08 | 0.61±0.12 | | 0.65±0.14 | 0.74±0.23 |
| Free fatty acids (mmol/L) | | 0.12±0.02 | 0.13±0.02 | 0.16±0.01 | 0.19±0.06 | | 0.15±0.02 | 0.12±0.02 | 0.20±0.05 | | 0.16±0.03 | 0.27±0.19 |
| ApoA-I (g/L) | | 0.89±0.05 | 0.81±0.03 | 0.74±0.08 | 0.80±0.03 | | 0.84±0.02 | 0.91±0.12 | 0.89±0.09 | | 0.87±0.05 | 0.90±0.40 |
| ApoB-100 (mg/L) | | 0.33±0.05 | 0.23±0.02 | 0.35±0.08 | **1.20±0.02 * †** | | 0.27±0.02 | 0.24±0.04 | 0.30±0.06 | | 0.24±0.02 | 0.39±0.15 |
|  | |  |  |  |  | |  |  |  | |  |  |

Data are organized according to maternal body mass index (BMI) at first trimester of pregnancy and weight gain during pregnancy (GWG). Results are expressed as mean ± SEM. ***** Groups statistically different (p<0.01) from the normal control group (BMI 20-26 kg/m^2^, GWG 11-18 kg) for a given parameter. **†** Groups statistically different (p<0.01) from the corresponding normal GWG group (11-18 kg) of similar BMI for a given parameter. All groups presenting differences are in bold characters.
