# Supplementary material for: Modulation of Apolipoprotein D levels in human pregnancy and association with gestational weight gain
Source: Reprod Biol Endocrinol. 2009 Sep 2;7:92. doi: 10.1186/1477-7827-7-92 (PMC3224896; doi:10.1186/1477-7827-7-92)
Supplement: Additional file 3 — Table 6: Association of plasma ApoD levels with clinical and biochemical characteristics in women at delivery. The data provided represent the Pearson correlations coefficients (r) between plasma ApoD levels and maternal characteristics at delivery. [file 1477-7827-7-92-S3.doc]

**Table 6: Association of plasma ApoD levels with clinical and biochemical characteristics in women at delivery.**

|  |  | | |  | | |  | | |
| --- | --- | --- | --- | --- | --- | --- | --- | --- | --- |
|  | BMI < 20 kg/m^2^ | | | BMI 20-26 kg/m^2^ | | | BMI > 26 kg/m^2^ | | |
|  | GWG < 11 kg  n= 9 | GWG 11-18 kg  n= 14 | GWG > 18 kg  n= 4 | GWG < 11 kg  n= 28 | GWG 11-18 kg  n= 48 | GWG > 18 kg  n= 6 | GWG < 11 kg  n= 20 | GWG 11-18 kg  n= 18 | GWG > 18 kg  n=4 |
|  |  |  |  |  |  |  |  |  |  |
| Gestational age (weeks) | **-0.229 *** | **0.404 **** | 0.326 | 0.086 | 0.079 | **-0.409 *** | **-0.280 **** | 0.070 | -0.899 |
| BMI  (kg/m^2^) | **0.284 *** | -0.046 | 0.984 | 0.160 | -0.115 | -0.326 | **-0.233 *** | -0.143 | 0.035 |
| GWG  (kg) | **0.675 *** | **-0.365 **** | -0.602 | **0.275 **** | -0.026 | -0.249 | **0.294 *** | **-0.253 ***** | -0.493 |
| Newborn weight (g) | -0.358 | 0.414 | -0.694 | 0.086 | 0.070 | -0.136 | 0.155 | 0.224 | 0.092 |
| Newborn height (cm) | 0.199 | 0.134 | -0.472 | -0.044 | -0.104 | -0.086 | 0.173 | 0.197 | 0.092 |
| Cord blood ApoD  (mg/L) | 0.761 | 0.784 | 0.478 | 0.171 | 0.204 | -0.169 | -0.074 | 0.056 | 0.018 |
|  |  |  |  |  |  |  |  |  |  |
| Total cholesterol (mM) | -0.561 | -0.064 | -0.589 | **0.202 ***** | **0.253 ***** | -0.548 | **0.272 ***** | 0.052 | 0.149 |
| LDL-cholesterol (mM) | -0.080 | -0.899 | -0.899 | **0.226 ***** | 0.179 | -0.739 | 0.107 | **0.378 ***** | -0.961 |
| HDL-cholesterol (mM) | 0.466 | -0.165 | -0.262 | 0.146 | -0.146 | -0.744 | 0.086 | **0.201 ***** | -0.996 |
| Triglycerides (mM) | -0.648 | 0.036 | 0.144 | **-0.214 ***** | 0.080 | 0.822 | **0.335 ***** | 0.163 | 0.997 |
| Free fatty acids (mmol/L) | **-0.346 *** | **-0.416 *** | -0.589 | -0.031 | 0.077 | -0.462 | -0.123 | **0.266 ***** | 0.534 |
| ApoA-I  (g/L) | -0.385 | **-0.200 **** | -0.742 | 0.093 | 0.127 | -0.582 | 0.018 | **-0.289 ***** | 1.00 |
| ApoB-100 (mg/L) | -0.642 | -0.038 | -0.776 | **0.332 ***** | 0.126 | 0.355 | **0.205 ***** | **0.335 ***** | -0.178 |
|  |  |  |  |  |  |  |  |  |  |

Pearson correlations (*r*) are significant at * p<0.05, ** p<0.05, ***p<0.001.
